# The nuclear factor (erythroid-derived 2)-like 2 (Nrf2) activator dh404 protects against diabetes-induced endothelial dysfunction

**DOI:** 10.1186/s12933-017-0513-y

**Published:** 2017-03-03

**Authors:** Arpeeta Sharma, Luddwi Rizky, Nada Stefanovic, Mitchel Tate, Rebecca H. Ritchie, Keith W. Ward, Judy B. de Haan

**Affiliations:** 1 Oxidative Stress Laboratory, Basic Science Domain, Baker Heart and Diabetes Institute, 75 Commercial Road, Melbourne, VIC 3004 Australia; 2 Heart Failure Pharmacology, Basic Science Domain, Baker Heart and Diabetes Institute, 75 Commercial Road, Melbourne, VIC 3004 Australia; 3 Reata Pharmaceuticals Inc., 2801 Gateway Dr, Irving, TX 75063 USA

**Keywords:** Endothelial dysfunction, hyperglycemia, Nrf2 activators, oxidative stress, inflammation, dh404, bardoxolone methyl

## Abstract

**Background:**

Vascular dysfunction is a pivotal event in the development of diabetes-associated vascular disease. Increased inflammation and oxidative stress are major contributors to vascular dysfunction. Nrf2, a master regulator of several anti-oxidant genes and a suppressor of inflammatory NF-κB, has potential as a target to combat oxidative stress and inflammation. The aim of this study was to investigate the effects of a novel Nrf2 activator, the bardoxolone methyl derivative dh404, on endothelial function in vitro and in vivo.

**Methods:**

dh404 at 3 mg/kg was administered to male Akita mice, an established diabetic mouse model of insulin insufficiency and hyperglycemia, from 6 weeks of age. At 26 weeks of age, vascular reactivity was assessed by wire myography, pro-inflammatory expression was assessed in the aortas by qRT-PCR and immunohistochemistry, and systemic and vascular oxidative stress measurements were determined. Additionally, studies in human aortic endothelial cells (HAECs) derived from normal and diabetic patients in the presence or absence of dh404 included assessment of pro-inflammatory genes by qRT-PCR and western blotting. Oxidative stress was assessed by three methods; L-012, DCFDA and amplex red. Static adhesion assays were performed to determine the leukocyte–endothelial interaction in the presence or absence of dh404.

**Results:**

Dh404 significantly attenuated endothelial dysfunction in diabetic Akita mice characterized by reduced contraction in response to phenylephrine and the downregulation of inflammatory genes (VCAM-1, ICAM-1, p65, IL-1β) and pro-oxidant genes (Nox1 and Nox2). Furthermore, reduced systemic and vascular oxidative stress levels were observed in diabetic Akita mice. dh404 exhibited cytoprotective effects in diabetic HAECs in vitro, reflected by significant upregulation of Nrf2-responsive genes, NAD(P)H quinone oxidoreductase 1 (NQO1) and heme oxygenase-1 (HO-1), reduction of oxidative stress markers (O_2_^·−^ and H_2_O_2_), inhibition of inflammatory genes (VCAM-1 and the p65 subunit of NF-κB) and attenuation of leukocyte–endothelial interactions (*P* < 0.05 for all in vitro and in vivo parameters; one or two-way ANOVA as appropriate with post hoc testing).

**Conclusion:**

These studies demonstrate that upregulation of Nrf2 by dh404 represents a novel therapeutic strategy to limit diabetes-associated vascular injury.

**Electronic supplementary material:**

The online version of this article (doi:10.1186/s12933-017-0513-y) contains supplementary material, which is available to authorized users.

## Background

A consistent phenomenon in diabetic patients is the development of endothelial dysfunction, which precedes the development of diabetes-associated vascular complications. Diabetic-endothelial dysfunction is characterized by enhanced vascular contractility, increased oxidative stress and vascular inflammation, in particular adhesion of inflammatory cells to the vascular wall [1–3]. Hyperglycemia-induced oxidative stress plays a critical role in accelerating processes that contribute to endothelial dysfunction. For example, elevated oxidative stress has been shown to reduce the bioavailability of the potent endothelial mediator, nitric oxide, resulting in vascular impairment [4]. In addition, oxidative stress directly elevates the expression of pro-inflammatory and adhesion molecules, facilitating the recruitment and infiltration of leukocytes/monocytes into the vasculature [5–7]. Despite the unequivocal evidence that supports the role of oxidative stress in diabetes-associated vascular complications, strategies to combat oxidative stress using traditional antioxidants have proved inconclusive in large clinical trials with respect to cardiovascular end-points [8]. Thus, current research is focused on mechanism-based antioxidants that offer a more targeted approach to ameliorate oxidative stress.

One such strategy that has gained considerable attention is the NF-E2-related factor 2 (Nrf2) pathway. Nrf2 is a ubiquitously expressed redox sensitive transcription factor that is kept in a latent state through its interaction with its repressor protein, Kelch ECH associating protein 1 (Keap-1). In response to increased free radicals, the Keap protein is oxidized or covalently modified releasing Nrf2, which then enters the nucleus and binds to the antioxidant response element (ARE), thereby initiating the transcription of a host of antioxidant genes, including superoxide dismutase (SOD), catalase, heme-oxygenase 1 (HO-1) and NAD(P)H:Quinone Oxidoreductase 1 (NQO1) [9–13]. Additionally, Nrf2 has demonstrated anti-inflammatory properties driven through its ability to negatively regulate nuclear factor-kappaB (NF-kB), the transcription factor central to the inflammatory response [14].

Numerous chemically diverse activators of the Nrf-2/Keap-1 pathways have been identified, including the natural compounds sulforaphane from cruciferous vegetables and cinnamic aldehyde found in cinnamon bark [13–15]. Synthetic small molecule activators of the Nrf2/Keap1 pathway include bardoxolone methyl (BM), which belongs to the antioxidant inflammation modulator (AIM) drug class [16–18]. BM has shown structural and functional improvements in rodent models of renal disease [19, 20], and its therapeutic potential was extended to clinical trials in type 2 diabetic patients with chronic kidney disease in which improvements in estimated glomerular filtration rate (eGFR) were observed, but with limitations, mainly concerned with patient selection, due to fluid overload complications [21–24]. Follow up analysis of the terminated BEACON trial has revealed important risk factors that need to be taken into account to avoid fluid overload and heart failure, including patients with elevated measures of baseline brain natriuretic peptide, a marker for fluid overload, and prior hospitalization for heart failure [25]. In a recently published study by our group, we demonstrated that a BM tool compound, dh404, attenuated atherosclerosis in diabetic Apolipoprotein E knockout (ApoE KO) mice in an inverse dose-dependent manner with lower doses being atheroprotective, highlighting the importance of careful dosage considerations for Nrf2 activators. Importantly, the antioxidant and anti-inflammatory properties of dh404 correlated with the observed improvements in diabetes-associated atherosclerosis [26]. Since endothelial dysfunction, which is the critical first step to the development of vascular complications, is accompanied by pro-oxidative and pro-inflammatory conditions, we hypothesized that dh404 might be exerting its atheroprotective effects via improvements in endothelial dysfunction in the diabetic setting. This aspect has not been studied previously and warrants further attention.

In the current study, we investigated the effects of the Nrf2 activator, dh404, on endothelial function in the Akita mouse, a model of hyperglycemia and hypoinsulinemia due to improper folding of proinsulin [27] and in cultured aortic endothelial cells isolated from diabetic patients. We show that Nrf2 activation via dh404 improves vascular function, by targeting pro-oxidative and pro-inflammatory pathways. As such, we propose that recovery of endothelial function, lost due to diabetes, leads to vascular protection, and limits diabetes-associated atherosclerosis.

## Methods

### Animal groups and experimental design

All animal experiments were approved by the Alfred Medical Research and Education Precinct (AMREP) animal ethics committee and investigations conformed to National Health and Medical Research Council (NHMRC; Australia) guidelines. Diabetic Akita mice (C57BL/6J-Ins2Akita) and their wild type (WT) counterparts were purchased from the Jackson Laboratory (Sacramento, CA, USA) and bred at the AMREP Precinct Animal Centre. At 6 weeks of age, male mice of both genotypes were randomly assigned to receive either vehicle (sesame oil), or dh404 (3 mg/kg) by daily oral gavage. As previously reported [26], this dose of dh404 is optimal in reducing diabetes-associated atherosclerosis. Blood glucose and body weights were recorded on a weekly basis as part of animal monitoring.

### Blood sampling, plasma biochemistry and tissue collection

At 26 weeks of age, mice were fasted for 3 h before termination for organ collection. Mice were anesthetized by injecting 2,2,2-Tribromoethanol (Avertin^®^; Sigma-Aldrich, St. Louis, MO, USA) intraperitoneally. Following which, the thoracic cavity was incised and blood was collected via direct puncture of the right ventricle. The blood was placed in heparinized tubes and centrifuged (room temperature for 10 min at 4000 rpm) to obtain plasma samples. Plasma glucose, cholesterol, HDL and triglycerides were measured as described previously [28].

The heart and aorta were dissected and placed in Krebs buffer (components in mM; NaCl 119, KCl 4.7, NaHCO3 25, MgSO4·7H20 1.17, CaCl2 2.5, KH2PO4 1.18, glucose 11 and EDTA 0.03). The aorta was cleaned to remove peripheral fat under a dissecting microscope and cut into two sections. The thoracic aorta was mounted on a wire myograph for vascular reactivity analysis while the arch and abdominal aorta was snap frozen in liquid nitrogen for RNA extraction. A portion of the thoracic aortas was frozen in OCT for immunohistochemical analysis.

### Vascular reactivity

Vascular reactivity studies were performed as per Vaisman et al. [29]. Briefly, the thoracic aorta was cut into 4 mm segments and mounted on two L-shaped metal prongs. One prong was linked to a force–displacement transducer for continuous recording of isometric tension by the LabChart software, while the other prong was connected to a displacement device, which allowed adjustment of the distance between the two parallel prongs. Aortas were then equilibrated for 30 min and normalised at resting tension (0 mN), 15 and 25 mN to obtain the final micrometer setting between the prongs.

Thereafter, aortas were exposed to an oxygenated and pre-warmed high potassium physiological salt solution (KPSS at 37 °C; components in mM; KCl 123, MgSO4·7H20 1.17, NaHCO3 25, KH2PO4 1.18, CaCl2 2.5, glucose 6.05 and EDTA 0.03) to determine the viability of the aortas. A dose–response to phenylephrine (PE) (1 nM–100 µM) was performed to assess vascular contractility and l-NG-Nitroarginine Methyl Ester (l-NAME; 100 μM)-induced contraction was assessed in aortas preconstricted to approximately 20% contraction of KPSS with u41699, an endothelium-independent vasoconstrictor agent. The variable slope sigmoidal concentration-responses curves to all agonists in each subject were calculated and plotted using GraphPad Prism (v6.0).

### Aortic RNA extraction, gene expression and immunohistochemistry analysis

Aortas were snap frozen and total RNA was extracted after homogenization as described previously [26]. Probes and primers were purchased from Applied Biosystems (ABI, Foster City, CA, USA). Gene expression of vascular cell adhesion molecule (VCAM-1), intracellular adhesion molecule (ICAM-1), nuclear factor (NF)-κB subunit p65, NAD(P)H oxidase (Nox) subunit 1 (Nox1), Nox2 and interleukin-1β (IL-1β) were analyzed by quantitative RT-PCR as described previously [26]. Frozen aortic sections were stained with VCAM-1 as described previously [26]. In brief, frozen aortic sections were fixed with cold acetone, and endogenous peroxidases were inactivated with 3% H_2_O_2_ in Tris-buffered saline. Sections were then incubated with a protein blocking agent and a biotin-avidin blocking kit (Vector Laboratories). Following which, aortic sections were incubated with VCAM-1 primary antibody (1:100) overnight at 4 °C. Biotinylated anti-rat immunoglobulin (1:200; Vector Laboratories) was then added for 30 min, followed by horseradish peroxidase–conjugated streptavidin, diluted 1:500 (Dako), and incubated for 30 min in 3,3′-diaminobenzidine tetrahydrochloride (Sigma-Aldrich) with hematoxylin counterstain. Images were visualized under light microscopy and quantitated using Image Pro Plus. Three to five sections were assessed per mouse and averaged to obtain a single value per mouse, and seven to nine mice were analyzed per group.

### Systemic oxidative stress and aortic superoxide measurements

Derivatives of reactive oxygen metabolites (dROMs) were measured in plasma as an indication of oxidative stress using the FRAS-4 system as described previously [26, 30]. In addition, urinary 8-isoprostanes, a marker for lipid peroxidation, was measured using an ELISA kit from Oxford Biomedical Research as per manufacturer’s instructions. Superoxide content in frozen aortic sections was measured using dihydroethidium (DHE; 10 μmol/L) staining and imaged using a Zeiss 510 Meta confocal microscope equipped with a krypton/argon laser (excitation 488 and emission 543 nm) as previously described by our group [31].

### Cell culture

Human aortic endothelial cells (HAECs) and HAECs isolated from a diabetic patient was purchased from Lonza Clonetics (CC-2919) and maintained in EGM-2 media (Lonza) supplemented with 10% FBS at 37 °C in 5% CO_2_. Experiments were performed on cells from passages 3–9. Upon 85% confluency, HAECs were treated with dh404 (25–100 nM) for a period of 24 h before experiments were performed.

### RNA extraction and RT-PCR of human aortic endothelial cells

Dh404-treated normal and diabetic HAECs were stimulated with TNF-α (1 ng/ml) for 2 h. Following which, cells were washed twice with ice-cold PBS and lysed with RNA lysis buffer containing DNAse enzyme. After an incubation period of 7 min at room temperature with gentle agitation, a stop solution was added and DNAase-treated RNA was collected and its concentration was determined using the nanodrop spectrophotometer at 260 and 280 nm. Following which, DNA-free RNA (3 µg) was reverse-transcribed into cDNA using the Superscript First Strand System. 50 ng/µl of random primers were first added to RNA samples and incubated at 70 °C for 5 min, followed by immediate placement on ice. Meanwhile, a master mix was made from 4 μL of 5X first strand buffer, 2 μL of 10 mM dNTPs, 2 μL of 0.1 M dithiothreitol, 0.1 μL of RNase inhibitor (Promega; 20 U/μL) and 1 μL of M-MuLV Reverse Transcriptase (200 U/μL). The master mix was then added to the RNA mixture and incubated at room temperature (25 °C) for 10 min and subsequently incubated at 37 °C for 60 min and at 70 °C for 10 min to complete the reverse transcription. The mixture was then pulse centrifuged to pellet any condensation and the cDNA was then stored at −20 °C until further use.

### Western blotting

HAECs were washed twice with PBS and protein was isolated using RIPA buffer as described previously [32]. Protein concentration was determined using the BCA protein kit. Protein lysates, containing equal amounts of protein, were electrophoresed on a TGX Precast gel (Bio-Rad). On completion, the gel was activated immediately and visualized on the BioRad imaging system for the detection and quantification of total protein using the Image Lab software. Proteins were then transferred to a polyvinylidene difluoride membrane using the trans-blot turbo transfer system (BioRad). Membranes were then blocked with 5% skim milk in TBS at room temperature for 1 h and then incubated with rabbit polyclonal VCAM-1 at 1:1000 dilution overnight at 4 °C. Pierce ImmunoPure Goat anti-rabbit IgG, peroxidase conjugated, was used as the secondary antibody for 1 h at room temperature and finally, proteins were visualized using the ECL Advance Western Blotting detection kit. Signals were quantitated by densitometry using Image Lab (Bio-Rad). Data are expressed relative to total protein and three independent experiments were analysed.

### In vitro leukocyte-adhesion assays

Monocyte–endothelial interactions were determined by performing in vitro static cell adhesion assays as described previously [33]. Briefly, normal and diabetic HAECs were treated with TNF-α (1 ng/ml; 4 h) in the presence or absence of dh404 (24 h pre-treatment). In the meantime, human monocytic THP-1 cells were labeled with the CellVue Burgundy fluorescent labeling kit (Affymetrix) as per the manufacturer’s instructions and incubated with treated HAECs for 20 min at 37 °C. Thereafter, HAECs were washed twice with phosphate-buffered saline, fixed with 10% NBF and plates were scanned using an Odyssey infra-red scanner. Fluorescence intensity (700 nm) of adherent THP-1 cells was quantified using Odyssey software.

### Determination of reactive oxygen species levels

Dh404-treated control and diabetic HAECs were incubated in phosphate buffered saline (PBS; with Ca^2+^/Mg^2+^) containing Dichloro-fluorescein diacetate (DCFDA; 5 µM), an intracellular probe to detect reactive oxygen species (ROS) [34] for 40 min at 37 °C. Following which, the cells were washed with PBS and incubated with PBS alone (control) or TNF-α (2 ng/ml) and placed into the Omega Fluorstar reader for fluorescence detection at 485 nm excitation and 530 nm emission. For L-012 assays, a chemiluminescent probe to detect superoxide [35], dh404-treated control and diabetic HAECs were incubated with Krebs buffer containing L-012 (20 μM) in the presence and absence of TNF-α (2 ng/ml) and placed in a luminometer to detect L-012 enhanced chemiluminescence for 1 h. Background readings were subtracted and relative chemiluminescence was quantified at 30 and 60 min. Amplex red for the quantification of hydrogen peroxide [36] was determined in dh404-treated control and diabetic HAECs using the Amplex Red Hydrogen Peroxide/Peroxidase Assay Kit (Molecular Probes) as per the manufacturer’s instructions.

### Statistical analysis

All data are expressed as mean ± standard error of mean (SEM). Comparison between groups were analysed by performing a two-way ANOVA with Tukey’s multiple comparison post hoc test. For vascular reactivity studies, a one-way ANOVA with Tukey’s multiple comparison post hoc test of the Rmax and EC_50_ values were performed. All statistical analyses were performed using GraphPad Prism version 6.0 (GraphPad Software, La Jolla, CA, USA). A *P* value <0.05 was considered statistically significant. Data and statistical analysis comply with the recommendations on experimental design and analysis in pharmacology [37].

## Results

### Body weight and metabolic parameters in WT and Akita mice

The body weight and metabolic parameters at the end point of the study of diabetic Akita mice and their WT counterparts treated with either vehicle or dh404 are depicted in Table 1. Akita mice displayed significantly lower body weights as compared to WT mice (p < 0.0001; Table 1). Treatment with dh404 did not affect the body weights in both WT and Akita mice when compared with vehicle controls (Table 1). Akita mice had significantly increased blood glucose and HbA1c levels as compared to WT mice, which was not altered with dh404 treatment (Table 1; p < 0.0001). Furthermore, the total cholesterol, triglycerides, HDL, and LDL levels were not significantly different between each group (Table 1). Overall, these findings indicate that treatment with dh404 did not alter the glucose and lipid parameters in WT and Akita mice.

**Table 1:** Impact of the Nrf2 activator dh404 on metabolic parameters in WT and Akita mice

	WT + vehicle	WT + dh404	Akita + vehicle	Akita + dh404
Body weight (g)	32.3 ± 1.1	30.9 ± 1.1	21.5 ± 0.6****	21.9 ± 0.6****
Blood glucose (mmol/L)	12.9 ± 1.0	11.6 ± 1.2	40.5 ± 3.7****	39.0 ± 4.1****
HbA1c (%)	4.5 ± 0.1	4.9 ± 0.1	13.0 ± 0.3****	12.0 ± 0.7****
Cholesterol (mmol/L)	1.8 ± 0.1	1.9 ± 0.1	1.7 ± 0.1	1.8 ± 0.2
Triglycerides (mmol/L)	0.6 ± 0.1	0.2 ± 0.1	0.9 ± 0.5	1.0 ± 0.3
HDL (mmol/L)	1.4 ± 0.1	1.5 ± 0.1	1.0 ± 0.1	1.1 ± 0.1
LDL (mmol/L)	0.2 ± 0.1	0.4 ± 0.1	0.5 ± 0.1	0.5 ± 0.1
LH ratio	0.2 ± 0.1	0.3 ± 0.1	0.7 ± 0.3	0.6 ± 0.2

Body weight, blood glucose, Hb1Ac, cholesterol, tryglycerides, HDL, LDL, and LH ratio were measured at 26 weeks of age. Data are presented as ±SEM. (**** P < 0.0001 vs. WT + vehicle; n = 7/group)

### Dh404 improves vascular function and oxidative stress in diabetic Akita mice

**Fig. 1:**
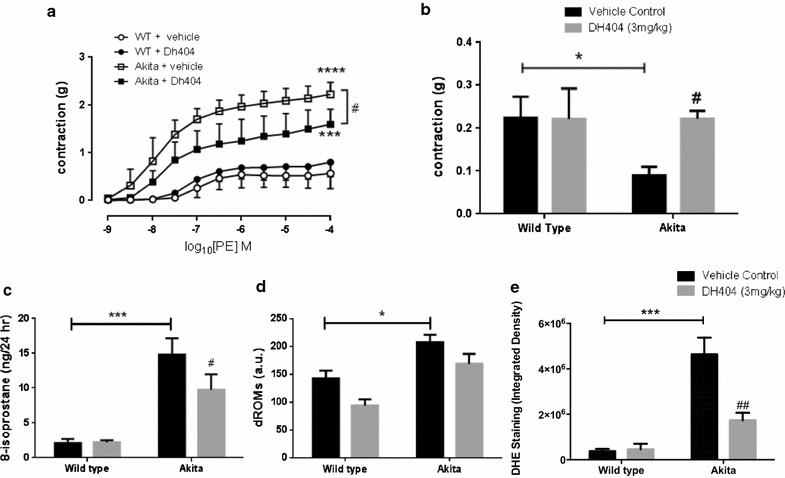
The Nrf2 activator dh404 improves vascular function in vivo. **a** Cumulative concentration–response curves to PE (0.001–100 µmol) and **b** contraction to l-NAME (100 µmol) was measured. *P < 0.05, ***P < 0.001 and ****P < 0.0001 versus WT + vehicle. ^#^P < 0.05 versus Akita + vehicle. **c** Urinary 8-isoprostane (ng/24 h) and **d** plasma dROMs were measured. **e** Quantification of superoxide detection by DHE fluorescence imaging in aortas of WT and Akita mice treated with vehicle or Dh404 (3 mg/kg). *P < 0.05 and ***P < 0.001 as indicated. ^#^P < 0.05 and ^#^P < 0.01 versus WT + vehicle. Data are presented as ±SEM. N = 4–8 per group

Endothelial function in the diabetic vasculature is markedly impaired due to increases in oxidative stress and inflammation. Therefore, we investigated if the Nrf2 activator dh404 can improve endothelial dysfunction in the diabetic milieu by measuring vascular contractility in aortic rings isolated from WT and Akita mice. Importantly, Akita aortas exhibited significantly enhanced contraction to PE as compared to the WT aortas, which indicates that there is diminished inherent vasodilator capacity possibly due to lowered NO levels to counteract the PE-constricting effect (Fig. 1a; P < 0.001). Although dh404 had no effect on WT aortas, dh404 was able to significantly reduce the enhanced PE contractility in Akita aortas (Fig. 1a; P < 0.05).

To confirm these findings, we added l-NAME, a NOS inhibitor, after a sub-minimal preconstriction with U46619, a thromboxane A_2_ analogue. l-NAME inhibits any endogenous eNOS-derived NO present in the system. l-NAME caused a further contraction in the WT aortas as compared to the vehicle-treated Akita group (Fig. 1b; P < 0.05), which is indicative of significantly more NO bioavailability in the WT groups. Importantly, dh404 was able to restore the contraction in the Akita group back to the levels observed in their WT counterparts (Fig. 1b; P < 0.05).

Additionally, we measured systemic ROS levels using two distinct assays, namely the urinary 8-isoprostane assay and plasma dROMs analysis. Urine from Akita mice displayed significantly elevated 8-isoprostane levels compared to WT mice urine (Fig. 1c; P < 0.001), which was significantly attenuated with dh404 treatment (Fig. 1c; P < 0.05). Similarly, plasma from Akita mice showed increased dROMs levels compared to plasma from WT mice (Fig. 1d; P < 0.05). Furthermore, dh404 treatment showed trends towards reduced plasma dROMs levels in both WT and Akita mice (Fig. 1d). Lastly, superoxide-specific DHE staining of aortic vessels from Akita mice showed a 12-fold increase as compared to WT vessels (Fig. 1e; P < 0.001). Whilst DH404 treatment had minimal effect on superoxide-specific staining in WT vessels, it significantly reduced aortic superoxide levels in Akita vessels (Fig. 1e; P < 0.01). Representative DHE stained vessels in the pressence and absence of Tempol are shown in Additional file 1: Figure S1

### Dh404 reduces pro-inflammatory and pro-oxidative markers in the diabetic Akita macrovasculature

**Fig. 2:**
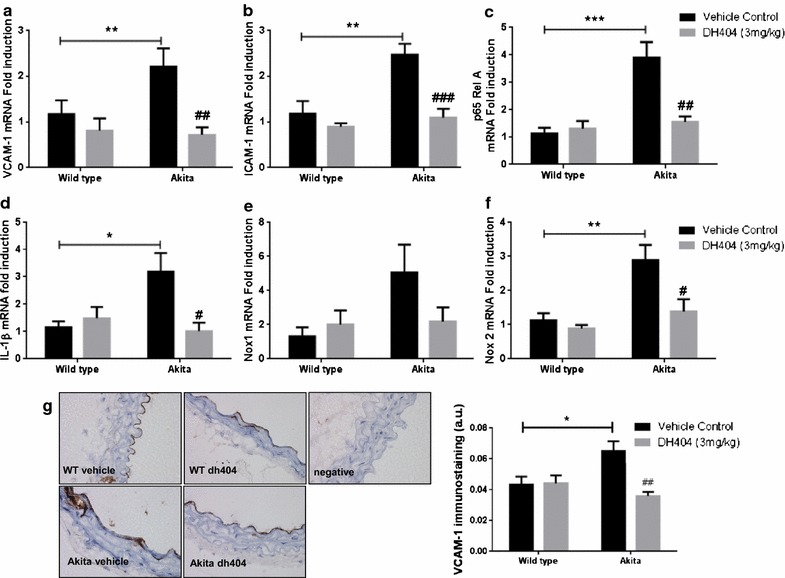
The Nrf2 activator dh404 reduces pro-inflammatory and pro-oxidative markers in diabetic Akita mice. mRNA expression levels of **a** VCAM-1, **b** ICAM-1, **c** p65 Rel A subunit of NF-κB, **d** IL-1β, **e** Nox1 and **f** Nox2 determined by qRT-PCR and expressed as fold induction relative to vehicle controls in WT mice. *P < 0.05, **P < 0.01 and ***P < 0.001 as indicated. ^#^P < 0.05, ^##^P < 0.01 and ^###^P < 0.001 versus Akita + vehicle. **g** Representative images and quantification (*right panel*) of VCAM-1 immunostaining in aortas of WT and Akita KO mice treated with vehicle or dh404. *P < 0.05 versus WT + vehicle group and ^##^P < 0.01 versus Akita + vehicle group. Data are presented as ±SEM. N = 6–8 per group

Inflammation and oxidative stress are major contributors towards the development of diabetes-associated endothelial dysfunction. Thus, we investigated the effect of dh404 on aortic gene expression levels of the pro-inflammatory markers, VCAM-1, ICAM-1, the p65 subunit of NF-κB and interleukin 1β (IL-1β), as well as the gene expression of the pro-oxidant enzyme Nox1 and Nox2. As expected, gene expression levels of these pro-inflammatory and pro-oxidant markers were elevated in diabetic Akita aortas as compared to WT aortas (Fig. 2a–f). dh404 had minimal effect on the expression of these genes in WT aortas. However, dh404 caused a significant reduction in pro-inflammatory and pro-oxidant gene expression in diabetic Akita aortas (Fig. 2a–f). This was further confirmed by investigating aortic VCAM-1 protein expression by immunohistochemistry. VCAM-1 expression was significantly increased in Akita aortas compared to WT aortas (Fig. 2g; P < 0.05). Dh404 reduced VCAM-1 protein expression in Akita aortas back to the levels observed in WT aortas (Fig. 2g; P < 0.01).

Collectively, these data demonstrate that Nrf2 activation by dh404 improves vascular function by enhancing vasodilation in diabetic Akita vessels and this is associated with reductions in vascular oxidative stress and inflammation.

### DH404 reduces oxidative stress parameters and upregulates antioxidant genes in diabetic settings

**Fig. 3:**
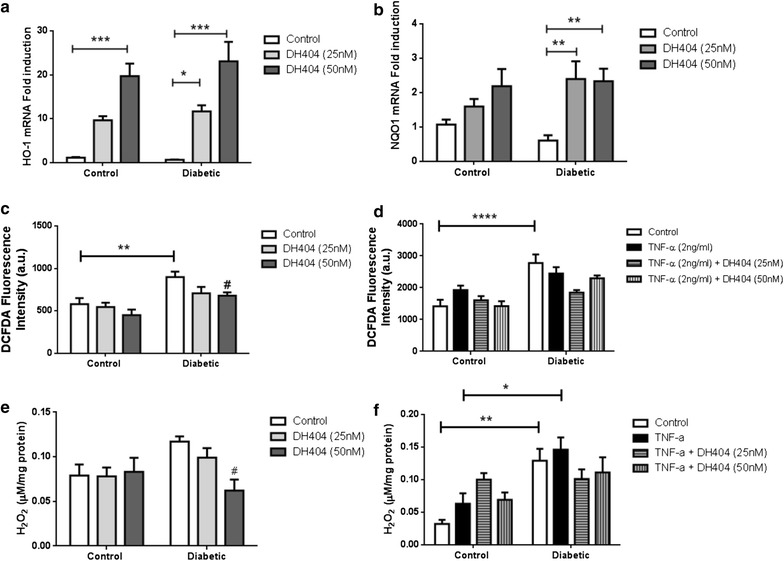
The effects of the Nrf2 activator dh404 on oxidative stress markers. Treatment with dh404 resulted in increased gene expression levels of HO-1 (**a**) and NQO1 (**b**). Dh404 treatment also resulted in attenuation of oxidative stress markers in basal and TNF-α treated control and diabetic HAECs. Oxidative stress markers are measured using DCFDA (**c** and **d**) and Amplex red (**e** and **f**) assays. Gene expression is relative to control HAECs. Data are presented as ±SEM. *P < 0.05, **P < 0.01, ***P < 0.001 and **** P < 0.0001 as indicated. ^#^P < 0.05 versus diabetic HAECs. A.U: arbitrary units. N = 6–8 per group

To specifically investigate the effects of Nrf2 activation of dh404 on endothelial cells, and to gain further mechanistic insight, we examined the effect of dh404 on oxidative stress parameters in control and diabetic HAECs. Firstly, to confirm that dh404 is acting via the Nrf2 pathway, we investigated the effect of dh404 treatment on downstream Nrf2 antioxidant responsive genes, in particular HO-1 and NQO1. The basal expression of both HO-1 and NQO1 genes were similar in control and diabetic HAECs (Fig. 3a, b). After treatment with dh404 (25–50 nM), there was a significant dose-dependent increase in HO-1 gene expression in both control and diabetic HAECs by ~20 and ~25 fold respectively (Fig. 3a; P < 0.001). Similarly, the expression of NQO1 was significantly increased following treatment with both doses in diabetic HAECs by ~2.5 fold as compared to their control (Fig. 3b; P < 0.01). Trends toward increased NQO1 gene expression was also observed in control HAECs after dh404 treatment (Fig. 3b). Next we measured hydrogen peroxide levels using two independent ROS assays (DCFDA and the Amplex Red assay). Basal hydrogen peroxide levels were elevated in diabetic HAECs as compared to control HAECs, as revealed by the DCFDA and Amplex Red assays (Fig. 3c–f). Furthermore, there appeared to be a dose dependent decrease in hydrogen peroxide levels after dh404 treatment in diabetic cells which became significant at the higher dose of 50 nM (Fig. 3c, e). A trend toward a decrease was noted in control cells probed with DCFDA in Fig. 3c. Treatment with TNF-α had a marginal effect on hydrogen peroxide levels, whilst co-treatment with dh404 showed trends towards a reduction, particularly in the diabetic setting (Fig. 3d, f). We also investigated the effect of dh404 on superoxide levels in control and diabetic cells after TNFα treatment in Additional file 2: Figure S2A. There was a dose dependent decrease in superoxide levels in control cells which was significant at the higher dose of 50 nM. Trends towards a decrease were noted in the diabetic cells at both concentrations of dh404. In general, these data highlight the protective antioxidant features of Nrf2 activation by dh404 through the elevation in antioxidant genes and the suppression of oxidative stress markers such as superoxide and hydrogen peroxide.

### Dh404 attenuates pro-inflammatory genes and leukocyte–endothelial interactions in vitro

**Fig. 4:**
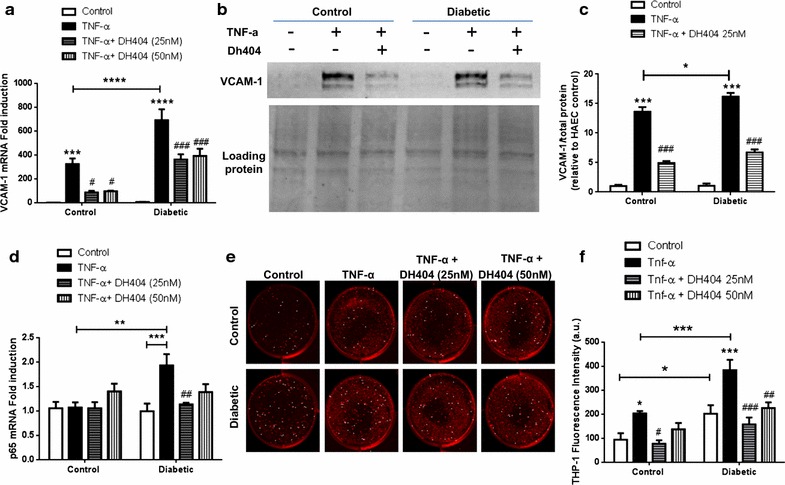
The Nrf2 activator dh404 inhibits pro-inflammatory markers and leukocyte–endothelial interactions in vitro. **a** VCAM-1 and **d** p65 gene expression is shown here. Gene expression is relative to control HAECs. **b** Representative VCAM-1 Western Blot and **c** quantification based on n = 3 blots. VCAM-1 is expressed based on loading protein for each sample and quantified relative to control HAECs. **e** Representative images and **f** quantification of adherent human THP-1 monocytic cells to control and diabetic HAECs treated with TNF-α and dh404. Data are presented as ±SEM. *P < 0.05, **P < 0.01, ***P < 0.001 and ****P < 0.0001 versus respective control treatments. ^#^P < 0.05, ^##^P < 0.01, ^###^P < 0.001 and ^####^P < 0.0001 versus respective TNF-α treated control and diabetic HAECs. N = 3–5 per group

Next, we examined the anti-inflammatory potential of dh404 in suppressing pro-inflammatory genes in control and diabetic HAECs. In particular, we investigated VCAM-1, an important pro-inflammatory marker that has been implicated in endothelial dysfunction and early stages of atherogenesis. The basal expression level of VCAM-1 is significantly elevated in diabetic HAECs by fivefold as compared to control HAECs (Additional file 2: Figure S2B). Upon TNF-α treatment, both control and diabetic HAECs showed significant increases in VCAM-1 expression. However, diabetic HAECs demonstrated a ~3 fold increase in VCAM-1 gene expression as compared to control HAECs (Fig. 4a; P < 0.0001). Importantly, VCAM-1 gene expression was significantly downregulated in response to dh404 treatment at both 25 and 50 nM in diabetic (Fig. 4a; P < 0.001) and control HAECs (Fig. 4a; P < 0.05). Given the significant increase in VCAM-1 gene expression, which was reduced with dh404, we investigated the effect of Nrf2 activation on VCAM-1 protein levels in vitro. A significant increase in VCAM-1 protein levels was observed in control and diabetic HAECs following TNF-α treatment as compared to untreated cells (Fig. 4b, c; P < 0.001). This increase was more pronounced in diabetic HAECs (Fig. 4b, c; P < 0.05). Treatment with dh404 significantly reduced VCAM-1 protein levels in both control and diabetic HAECs (Fig. 4b, c; P < 0.001). Next we evaluated the gene expression of the p65 subunit of NF-κB which is upstream of VCAM-1 induction. The expression of p65 was significantly increased in diabetic HAECs by up to ~2 fold compared to control HAECs following treatment with TNF-α (Fig. 4d; P < 0.01). Importantly, treatment with dh404 at 25 nM significantly suppressed p65 gene expression in diabetic HAECs back to the level observed in control HAECs (Fig. 4d; P < 0.01). Neither TNFα nor dh404 had any significant effect in control HAECs.

Furthermore, in unstimulated conditions, we observed that diabetic HAECs exhibited significantly more adhesion of fluorescently labelled monocytic THP-1 cells as compared to control HAECs by ~2 fold (Fig. 4e, f; P < 0.05). Addition of TNF-α induced a robust increase of THP-1 adhesion to both control and diabetic HAECs with significantly more attachment to the diabetic cells compared with controls (Fig. 4e, f; P < 0.05 and P < 0.001). Importantly, Nrf2 activation by dh404 significantly attenuated the leukocyte–endothelial interaction in both cell types back to levels observed in unstimulated conditions (Fig. 4e, f). In summary, these data suggest that dh404 transcriptionally regulates inflammatory processes, such as leukocyte–endothelial interactions in HAECs, in particular, by inhibiting the expression of p65 and its downstream mediator, VCAM-1.

## Discussion

Oxidative stress and inflammation have been interlinked as the underlying etiology in the pathogenesis of diabetes-associated endothelial dysfunction [38]. Strategies to ameliorate both oxidative stress and inflammation may be beneficial in improving diabetes-associated endothelial dysfunction. In the current study, we demonstrate that a potent activator of Nrf2, the synthetic compound dh404, improved endothelial dysfunction in a diabetic mouse model in vivo and attenuated oxidative stress and inflammatory markers in the setting of diabetes in vitro. Specifically, our findings showed that compromised endothelial function as defined by enhanced phenylephrine contractility, was improved after treatment with dh404 in a diabetic mouse model. Additionally, a range of pro-inflammatory mediators and oxidative stressors were lessened in response to dh404 treatment in the diabetic vasculature. Furthermore, the increased oxidative stress and pro-inflammatory markers detected in cultured endothelial cells from diabetic patients were attenuated by dh404 treatment. Importantly, the reduction in oxidative stress and pro-inflammatory mediators was associated with decreased adhesion of monocytes to endothelial cells in vitro. Collectively, our study shows that treatment with the Nrf2 activator, dh404, confers protection against diabetic endothelial dysfunction.

A characteristic phenomenon of vascular endothelial dysfunction is a deficiency in bioavailable vasodilators in particular nitric oxide (NO), which is reflected by hypersensitization to PE-induced vasoconstriction. We demonstrated that in comparison to aortic vessels isolated from WT mice, enhanced contraction to PE occurred in vessels from Akita mice, indicating that endogenous NO bioavailability is reduced in diabetic Akita vessels. These observations corroborate data from previous studies that show increased sensitivity to PE in vessels isolated from db/db and Akita diabetic mice [39, 40]. To confirm our hypothesis that endogenous NO bioavailability is compromised, we assessed LNAME-induced contraction. Since l-NAME inhibits eNOS function, further endogenous NO bioavailability is compromised such that the observed contraction induced by l-NAME is a reflection of the amount of endogenous NO present. Akita mice exhibited lower NO levels reflected by less LNAME-induced contraction. Our study shows that Dh404 treatment blunts PE- and l-NAME-induced contraction, indicating improved endothelial function by dh404 in diabetic aortas, which is in agreement with the study by Aminzadeh et al. [41] which demonstrated that dh404 improved CKD-induced endothelial dysfunction in rats associated with heightened level of oxidative stress.

Mechanistically, our results with dh404 showing an increase in Nrf2-responsive downstream genes as well as reductions in oxidative stress in a diabetic milieu, are in agreement with the known action of dh404 to act as an Nrf2 activator. Indeed, previous studies using adenovirus mediated overexpression of Nrf2 in endothelial cells resulted in marked upregulation of antioxidant responsive element (ARE)-driven genes in particular HO-1 and NQO1, protection against oxidative-stress mediated cytotoxicity and suppression of the inflammatory adhesion molecules, monocyte chemotactic protein-1 (MCP-1) and VCAM-1 [9, 42]. On the other hand, Nrf2 gene silencing demonstrated increased NF-κB activity and pro-inflammatory gene expression in cultured endothelial cells exposed to high glucose conditions [43]. Furthermore, the vascular endothelium of Nrf2 knockout mice displays a heightened pro-inflammatory and atherogenic state compared to wild-type mice [43]. Therefore, dh404 in its capacity to act as an AIM, has demonstrated improved endothelial dysfunction in this study via its ability to modulate anti-oxidant and pro-inflammatory pathways. Additionally, we demonstrate that dh404 affects pro-oxidant pathways since we show that the expression of prominent Nox family members is attenuated by dh404 in Akita vessels.

Endothelial oxidative processes are directly implicated in pathogenic changes in the vasculature which includes vascular remodelling and inflammation, particularly through the induction of inflammatory molecules. The role of VCAM-1, an important pro-atherosclerotic cellular adhesion molecule, has been shown to be regulated by the Nrf2 signaling pathway. Nrf2 negatively regulates VCAM-1 in the atheroprotected region of the aorta via modulating the p38-MAPK pathway [3]. Sulforaphane, a known Nrf2 activator, downregulates the expression of VCAM-1 in aortas of mice fed a high fat diet, a known model of type 2 diabetes. This upregulation of Nrf2 activity was associated with overall protection against structural vascular changes induced in the diabetic environment [15]. Our results are in alignment with these studies as we demonstrate a marked reduction in gene and protein expression of VCAM-1 after dh404 treatment in both our in vitro and in vivo diabetic models. This was associated with a decreased expression of the p65 subunit of NF-kB, an inflammatory transcriptional modulator. Furthermore, our study shows for the first time in a diabetic setting that upregulation of Nrf2 activity and subsequent downstream regulation of VCAM-1 is associated with less adhesion of circulating monocytes to endothelial cells in vitro, which suggests that targeting the Nrf2 pathway is beneficial in limiting leukocyte recruitment, a critical early step in atherogenesis. Our study is also consistent with earlier observations showing that AIMs such as RTA 403 or CDDO-Im suppress leukocyte adhesion in mouse retina [44]. However, it is imperative to note that the role of Nrf2 remains controversial in the development of cardiovascular diseases, particularly in atherogenic settings where Nrf2 has been shown to have opposing influences. Increases in Nrf2 expression have been reported to have an anti-inflammatory effect in athero-susceptible vascular regions, which is mediated mainly through its regulation of HO-1 [45, 46]. However, despite its anti-oxidative and anti-inflammatory actions, Nrf2 knockout mice on an ApoE−/− background exhibited protection against atherosclerosis. This was shown to be due to the role that Nrf2 plays in CD36 expression and ox-LDL uptake into macrophages, thereby promoting macrophage infiltration and foam cell formation in the vascular endothelium [47]. Due to the complexity of Nrf2 regulation in cardiovascular diseases it is apparent that therapeutic modulation of Nrf2 must consider the delicate balance between pro-inflammatory and anti-inflammatory actions of Nrf2. Indeed, as we have previously shown for Nrf2 activation, a window of atheroprotection exists above which the beneficial protective effects are lost [26], suggesting that careful dosage considerations are needed. Indeed, too much Nrf2 activation could disturb the homeostatic balance by blunting important ROS-based signalling with detrimental consequences. A further complexity arises when atherosclerosis is assessed in Nrf2−/− mice crossed with LDL R−/− mice, where in this instance lack of Nrf2 promoted atherogenesis through the increased formation of foam cells [48], suggesting that local interactions of Nrf2 may affect outcomes.

Finally, it is noteworthy that we observed significant differences between HAECs derived from diabetic versus non-diabetic patients with respect to basal levels of oxidative stress, pro-inflammatory mediators and pro-oxidant enzymes. Given that HAECs were derived from a diabetic patient yet cultured in low glucose, it is tempting to speculate that basal levels had become modified in favour of a more activated state whilst exposed to the diabetic environment within the patient, suggesting a memory effect. This is consistent with current theories where glucose-driven epigenetic modifications alter gene expression [49, 50]. This may also account for the legacy effect seen in clinical trials of intense glucose lowering [51]. However, one caveat needs to be kept in mind that the HAECs are derived from one control and one diabetic patient respectively and patient variability may play a significant factor.

## Conclusion

**Fig. 5:**
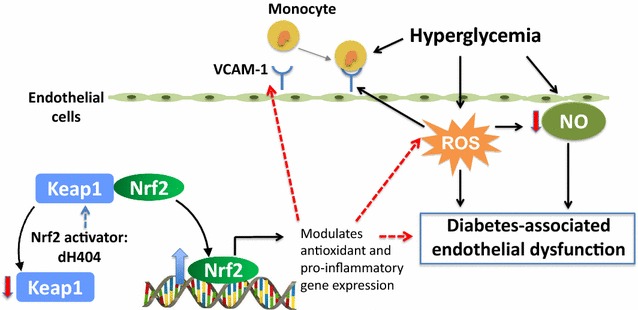
Graphical representation of the role of dh404 in limiting diabetes-associated endothelial dysfunction. Hyperglycemia increases ROS generation, lowers endothelial derived NO and upregulates inflammatory pathways, in particular leukocyte–endothelial interactions which are mediated by VCAM-1. Consequently there is a reduction in vascular function, a phenomenon described as diabetes-associated endothelial dysfunction. Dh404 is an Nrf2 activator, which disrupts the Nrf2/Keap-1 interaction, thereby allowing Nrf2 to translocate to the nucleus where it modulates antioxidant and pro-inflammatory gene expression. This study demonstrates that the Nrf2 activator, dh404, inhibits ROS production, lessens pro-inflammatory mediators and improves vascular function. In particular, we show that dh404 downregulates VCAM-1 which is associated with reduced leukocyte–endothelial interactions. *Black arrows* represent stimulation; *red dotted lines* represent inhibition

In conclusion, our study shows that upregulation of Nrf2 activity through pharmacological intervention is beneficial in ameliorating endothelial dysfunction in diabetic vascular disease settings. Given the critical role of maintaining a functional endothelium, our strategy has implications for the protection against diabetes-associated atherosclerosis, a diabetic complication with significant health burden. Mechanistically, we now show that Nrf2 upregulation with dh404 is associated with anti-inflammatory and anti-oxidative actions within diabetic aortic vessels and in human aortic endothelial cells. This translated into improved vascular contractility and reduced endothelial–leukocyte interactions in the diabetic milieu (Fig. 5). Overall, we speculate that Nrf2 activation represents a novel therapeutic strategy to improve diabetes-associated endothelial function which in turn may protect against diabetes-associated atherosclerosis. With the current greater understanding of which patients are likely to benefit from this class of drug based on follow-up analysis of the BEACON trial, which helped identify an important set of at-risk patients [25], further pre-clinical research into the cardiovascular outcomes of this drug class, such as this study, and that of other novel Nrf2 activators is warranted.

## Additional files

**Additional file 1: Figure S1.** Representative images of superoxide detection by fluorescence imaging of DHE (left panel) and DHE plus tempol, a superoxide mimetic (right panel) in aortas of WT and Akita mice in the presence and absence of dh404 (3 mg/kg).

**Additional file 2: Figure S2.** (A) Superoxide levels was measured in normal and diabetic HAEC in the presence of TNF-α using the L-012 assay. **P < 0.01 and ****P < 0.0001 versus respective control treatments. ^#^P < 0.05 versus respective TNF-α treated control HAECs. n = 6–8 per group. (B) Basal VCAM-1 expression in control and diabetic HAECs is shown here. Gene expression is relative to control HAECs. *P < 0.05 as indicated. Data are presented as ± SEM.

## Abbreviations

AMREP: Alfred Medical Research and Education Precinct
ApoE KO: apolipoprotein E knockout
ARE: antioxidant response element
AIM: antioxidant inflammation modulator
BM: bardoxolone methyl
DHE: dihydroethidium
DCFDA: dichloro-fluorescein diacetate
dROMs: derivatives of reactive oxygen metabolites
eGFR: estimated glomerular filtration rate
HAECs: human aortic endothelial cells
HO-1: heme-oxygenase 1
ICAM-1: intracellular adhesion molecule
IL-1β: interleukin-1β
Keap-1: Kelch ECH associating protein 1
l-NAME: l-NG-nitroarginine methyl ester
MCP-1: monocyte chemotactic protein-1
NF-kB: nuclear factor-kappaB subunit p65
NO: nitric oxide
Nox1: NAD(P)H oxidase subunit 1
Nox2: NAD(P)H oxidase subunit 2
NQO1: NAD(P)H quinone oxidoreductase 1
Nrf2: NF-E2-related factor 2
PE: phenylephrine
ROS: reactive oxygen species
SOD: superoxide dismutase
VCAM-1: vascular cell adhesion molecule
WT: wild type
