# Supplementary material for: The nuclear factor (erythroid-derived 2)-like 2 (Nrf2) activator dh404 protects against diabetes-induced endothelial dysfunction
Source: Cardiovasc Diabetol. 2017 Mar 3;16:33. doi: 10.1186/s12933-017-0513-y (PMC5335831; doi:10.1186/s12933-017-0513-y)
Supplement: Supplementary file 1 — Additional file 1: Figure S1. Representative images of superoxide detection by fluorescence imaging of DHE (left panel) and DHE plus tempol, a superoxide mimetic (right panel) in aortas of WT and Akita mice in the presence and absence of dh404 (3 mg/kg). [file 12933_2017_513_MOESM1_ESM.pptx]

## Slide 1
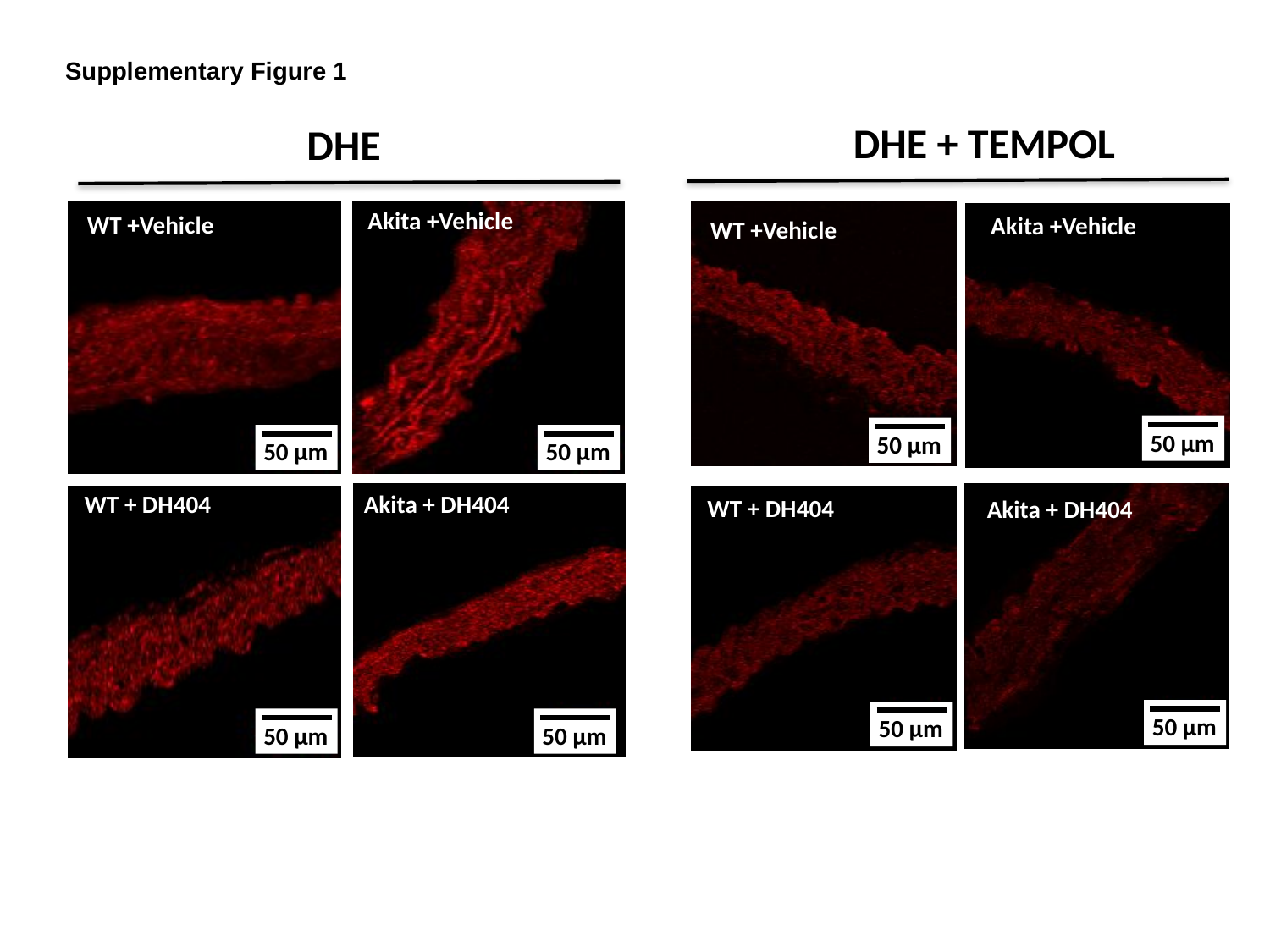

Supplementary Figure 1
DHE + TEMPOL
DHE
Akita +Vehicle
WT +Vehicle
Akita +Vehicle
WT +Vehicle
50 µm
50 µm
50 µm
50 µm
WT + DH404
Akita + DH404
WT + DH404
Akita + DH404
50 µm
50 µm
50 µm
50 µm
