# Supplementary figures and images for: The nuclear factor (erythroid-derived 2)-like 2 (Nrf2) activator dh404 protects against diabetes-induced endothelial dysfunction
Source: Cardiovasc Diabetol. 2017 Mar 3;16:33. doi: 10.1186/s12933-017-0513-y (PMC5335831; doi:10.1186/s12933-017-0513-y)

## Slide 1
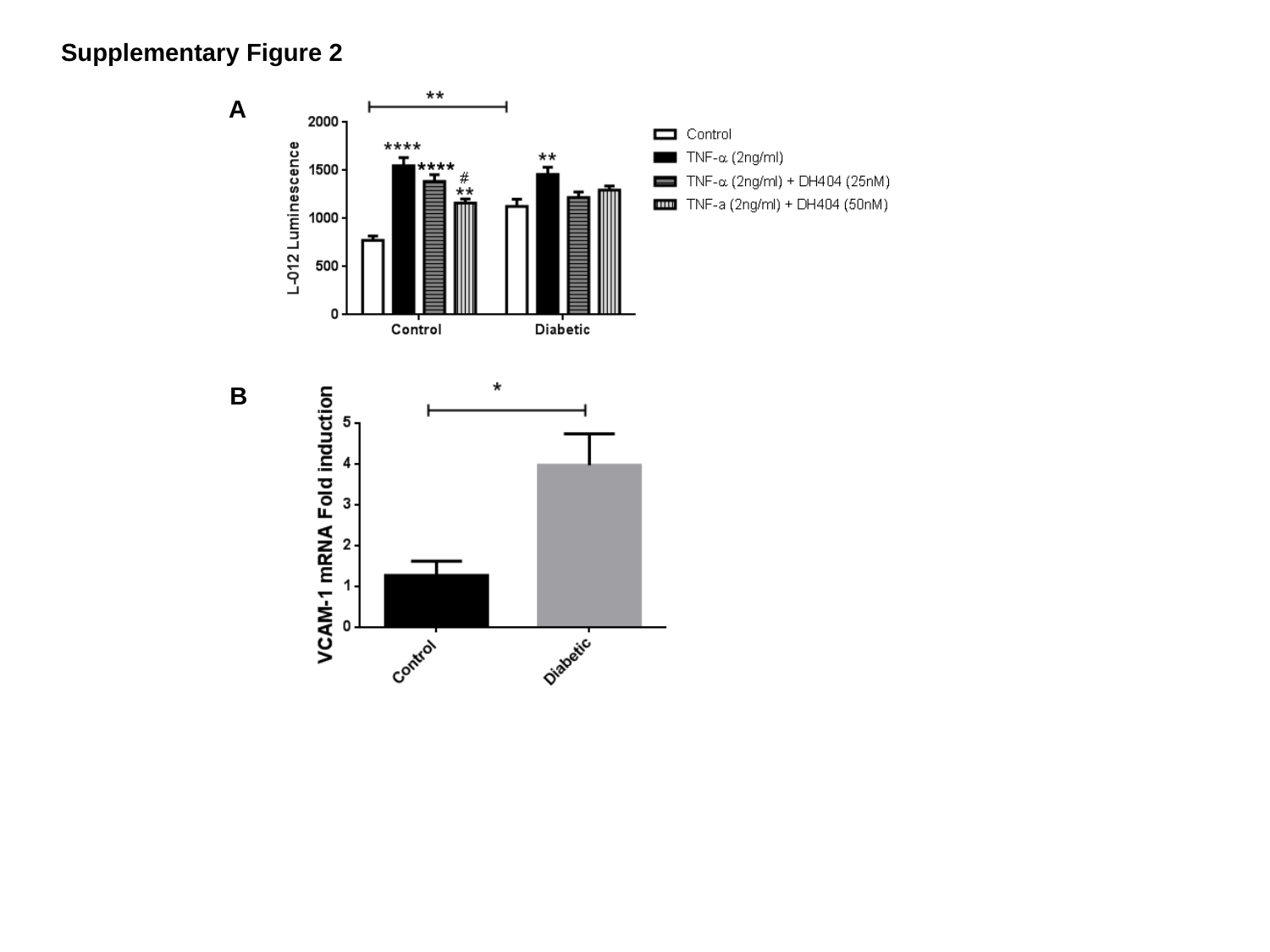

Supplementary Figure 2
A
B

Supplement: Supplementary file 2 — Additional file 2: Figure S2. (A) Superoxide levels was measured in normal and diabetic HAEC in the presence of TNF-α using the L-012 assay. **P < 0.01 and ****P < 0.0001 versus respective control treatments. #P < 0.05 versus respective TNF-α treated control HAECs. n = 6–8 per group. (B) Basal VCAM-1 expression in control and diabetic HAECs is shown here. Gene expression is relative to control HAECs. *P < 0.05 as indicated. Data are presented as ± SEM. [file 12933_2017_513_MOESM2_ESM.pptx]
